# Atypical Exon 2/3 Mutants G48C, Q43K, and E37K Present Oncogenic Phenotypes Distinct from Characterized NRAS Variants

**DOI:** 10.3390/cells13201691

**Published:** 2024-10-12

**Authors:** Mark Anthony G. Fran, Dominique Mickai G. Leaño, James Allen D. de Borja, Charles John T. Uy, Aleq Adrianne R. Andresan, Dennis L. Sacdalan, Reynaldo L. Garcia

**Affiliations:** 1 The Graduate School, Thomas Aquinas Research Complex, University of Santo Tomas, España, Manila 1008, Philippines; fran.biology@gmail.com; 2 Disease Molecular Biology and Epigenetics Laboratory, National Institute of Molecular Biology and Biotechnology, University of the Philippines Diliman, Quezon City 1101, Philippines; dgleano@up.edu.ph (D.M.G.L.); jddeborja1@up.edu.ph (J.A.D.d.B.); charles.uy@onjcri.org.au (C.J.T.U.); arandresan@up.edu.ph (A.A.R.A.); dlsacdalan1@up.edu.ph (D.L.S.); 3 Division of Medical Oncology, Department of Medicine, University of the Philippines, Manila City 1000, Philippines

**Keywords:** NRAS, young-onset colorectal cancer (YO-CRC), Ras isoform, epidermal growth factor receptor

## Abstract

NRAS belongs to the RAS family of GTPases. In colorectal cancer (CRC), NRAS mutations are rare compared to KRAS, but may lead to worse outcomes. We report the functional characterization of the novel NRAS mutants—G48C, Q43K, and E37K—identified in Filipino young-onset CRC patients. Unlike previously characterized NRAS mutants with no apparent effects on cell proliferation, these mutants enhanced proliferation of both HCT116 and NIH3T3 cells. This was confirmed in 3D spheroid assays to mimic the spatial organization of cells. G48C and E37K showed apoptosis resistance in both cell lines, and Q43K showed resistance in HCT116 cells. All three showed no effect on cellular migration in NIH3T3, but G48C enhanced the migration rate of HCT116 cells. Actin staining of NIH3T3 cells expressing the mutants showed a shrunken cytoplasm and transient structures associated with motility and invasiveness. Docking simulations show that GDP is only able to bind fully within the binding pocket of wild-type NRAS, but not in the mutants. Further, G48C, Q43K, and E37K all have less negative ΔG values, indicating a weaker GDP-binding affinity compared to wild-type NRAS. Taken together, the results suggest that oncogenic readouts of NRAS mutants are codon- and mutation-specific, with potential repercussions on the aggressiveness, resistance, and therapeutic response.

## 1. Introduction

Cancer is a multifactorial disease characterized by various hallmarks that include, among others, uncontrolled cell proliferation, increased migratory capacity, and resistance to apoptosis. In the Philippines, colorectal cancer (CRC) stands as the fourth leading cause of cancer-related mortality. This figure continues to rise due to delayed detection, lack of a formal CRC screening program in the country, and the relative lack of attention it receives compared to breast and lung cancer [1]. On a global scale, it ranks third as the most common among the various cancer types, with an estimated 3.2 million cases projected by the year 2040 [2].

The epidermal growth factor receptor (EGFR) and its family of receptor tyrosine kinases play a crucial role in transmitting signals within a cell, initiating various cellular processes such as proliferation, survival, migration, differentiation, and the regulation of gene expression. The signaling cascade commences with the binding of epithelial growth factor (EGF) to EGFR, which sets in motion a sequential activation of downstream effectors, including RAS, RAF, MEK, and ERK. Among these, the RAS proteins stand out as pivotal regulators of cell growth. RAS belongs to a superfamily of small GTPases involved in cell signaling pathways [3]. It acts as a molecular switch that cycles between an active, GTP (guanosine triphosphate)-bound state, and an inactive, GDP (guanosine diphosphate)-bound state. It serves as a key mediator in transmitting signals from cell surface receptors to the cell’s interior, thereby influencing fundamental aspects of cell behavior and function. The three well-characterized Ras isoforms—H-RAS, K-RAS, and N-RAS—are frequently mutated and serve as crucial biomarkers in various cancer types [2,4,5]. Mutations in KRAS, NRAS, and BRAF, along with the amplification of ERBB2 and MET, contribute to initial (de novo) resistance to anti-EGFR therapy, and have also been found in patients who initially responded to the EGFR blockade but later had a relapse [6].

The non-redundant and overlapping functions of the three canonical RAS isoforms remain incompletely understood. These RAS isoforms are also preferentially mutated in specific tumor types [7]. KRAS mutations are more common in pancreatic adenocarcinomas [8], colorectal cancer [9], and non-small-cell lung cancer [10]. NRAS mutations are more common in hematological malignancies [11] and melanoma [12]. Mutant HRAS, on the other hand, is most common in dermatological and head and neck cancers [13]. Further, although analogous mutations are found in the same codon position among the isoforms, their frequency varies depending on the cancer type [14]. For instance, while codon 12 and codon 61 mutants are found in both KRAS and NRAS, codon 12 is more frequently mutated in KRAS, while codon 61 mutations are more frequent in NRAS [15].

NRAS is the less-studied isoform of RAS [16]. NRAS mutations occur in 3–5% of CRCs, some of which have been shown to affect prognosis in metastatic colorectal cancer (mCRC), and may predict a lack of response to anti-EGFR therapy [17,18]. For the latter reason, the European Medicines Agency does not recommend the use of anti-EGFR therapy in CRC patients harboring NRAS mutations [17]. Further, mutations in NRAS are linked to poorer survival and outcomes compared to both KRAS-mutant and wild-type mCRC [19].

In this study, we describe the effects of three novel NRAS mutations—c.142G>T (G48C), c.127C>A (Q43K), and c.109G>A (E37K)—on cellular phenotypes, identified in a prospective, targeted, next-generation sequencing study of tumor samples from Filipino young-onset, colorectal cancer (YO-CRC) patients [20]. Although mutations in codons 43 and 48 of NRAS have been entered in the COSMIC database (https://cancer.sanger.ac.uk), Q43K and G48C, specifically, have not been reported before. There are no reported mutations for codon 37 in the COSMIC database.

## 2. Materials and Methods

### 2.1. Generation of NRAS Mutant Constructs

The NRAS G48C, Q43K, and E37K mutant constructs were generated using a wild-type *NRAS* construct as the template. G48C was generated via splicing by overlap extension (SOE) PCR (polymerase chain reaction) site-directed mutagenesis. First-round PCR was conducted using an external forward primer paired with an internal mutagenic reverse primer, and an internal mutagenic forward primer paired with an external reverse primer to generate the overlapping mutated left and right halves of *NRAS*, respectively. Aliquots of the two overlapping halves were combined, and they served as a template to generate the full-length construct using the external forward and external reverse primers. The full-length mutant amplicon was TA-cloned into the pTargeT™ mammalian expression vector (Promega Corporation, Madison, WI, USA). The Q43K and E37K constructs were generated using an inverse PCR site-directed mutagenesis approach, with overlapping mutagenic primers oriented in opposite directions, followed by circularization of the amplicon via the region of overlap. The primer sequences used are listed in Table 1.

The PCR cocktail contained 1× PCR buffer (from 5× SuperFi buffer; Invitrogen, Thermo Fisher Scientific, Waltham, MA, USA), 10 mM dNTP of each deoxynucleoside triphosphate (Promega), 10 µM each of the appropriate forward and reverse primers, 1× Platinum SuperFi DNA polymerase (Invitrogen), and 10 ng of template. PCR cycling was conducted using the C1000 Touch Thermal Cycler (BioRad Laboratories, Inc., Hercules, CA, USA). For SOE-PCR site-directed mutagenesis, the PCR reaction mix was initially denatured at 95 °C for 5 min, followed by 30 cycles of denaturation at 95 °C, annealing at 55 °C, extension at 72 °C—all aforementioned steps at 30 s each—and a final extension at 72 °C for 5 min. For inverse PCR site-directed mutagenesis, the PCR reaction mix was initially denatured at 98 °C for 30 s. This was followed by 30 cycles of denaturation at 98 °C for 10 s, annealing at 65 °C for 10 s, extension at 72 °C for 3 min, and a final extension at 72 °C for 5 min.

The wild-type *NRAS* and the canonical *NRAS* Q61K mutant constructs were previously cloned, and are available in the laboratory [21]. All *NRAS* constructs were sequence-verified. Expression of the wild-type and mutant NRAS proteins was confirmed via Western blot analysis (Supplementary Figure S1), following the protocol described below. The NRAS antibody used (rabbit α-NRAS polyclonal antibody, Cat. No. PA5-34560; Invitrogen) easily detects the NRAS protein from overexpression constructs. Its ability to detect endogenous levels of NRAS may vary depending on multiple factors, but it can be sensitive under optimal conditions [22].

### 2.2. Cell Culture Maintenance and Transfection

Two cell lines were used in this study, as follows: the NIH3T3 murine embryonic fibroblast cell line (Cat. No. CCL-247) and the HCT116 human colorectal carcinoma cell line (Cat. No. CCL-247), which were both obtained from the American Type Culture Collection (Manassas, VA, USA). NIH3T3 has a wild-type background, and is a preferred and well-documented heterologous host for studying RAS mutations because of its ability to manifest oncogenic phenotypes upon sole expression of a transforming Ras variant, without requiring cooperative mutations [23,24,25,26,27]. HCT116 provides the colorectal and epithelial background, but, similar to other commonly used colorectal cancer cell lines with mutations in either KRAS or PIK3CA, or both, it may mask the phenotypic effects of introduced mutations [28,29].

NIH3T3 cells were maintained in Dulbecco’s modified Eagle’s medium (DMEM; Gibco, Grand Island, NE, USA), enriched with 10% newborn calf serum (NBCS; Gibco), 100 U/mL penicillin/streptomycin, and 3.7 g/L sodium bicarbonate. HCT116 cells were grown in Roswell Park Memorial Institute (RPMI) 1640 medium (Gibco, Thermo Fisher Scientific, Inc., Waltham, MA, USA), enriched with 10% fetal bovine serum (FBS; Gibco, Waltham, MA, USA), 50 U/mL penicillin/streptomycin, and 2.0 g/L sodium bicarbonate. Both cell lines were maintained in a humidified incubator at 37 °C, with 5% CO_2_.

To achieve 80–90% transfection, HCT116 cells were seeded into 12-well plates at 150,000 cells/well, while NIH3T3 cells were seeded in a separate set of 12-well plates at 100,000 cells/well. Both cell lines were transfected with 300 ng of pTargeT™ constructs using Lipofectamine^®^2000 (Invitrogen), following the manufacturer’s protocol. Parallel transfection with pmR-ZsGreen1 (Clontech Laboratories, Inc., Mountain View, CA, USA), a reporter vector that expresses the green fluorescent protein ZsGreen1, was included to assess transfection efficiency using a fluorescence microscope (ZOE™ Fluorescent Cell Imager, Bio-Rad Laboratories, Hercules, CA, USA) for 24 h post-transfection for all cell-based functional and molecular characterization assays.

### 2.3. Western Blot Analysis

HCT116 and NIH3T3 cells were harvested and lysed for protein extraction 48 h post-transfection. Radioimmunoprecipitation lysis buffer (150 mM NaCl, 1.0% IGEPAL^®^ CA-630, 0.5% sodium deoxycholate, 0.1% SDS, 50 mM Tris, pH 8.0) (Sigma-Aldrich Corp.; Merck KGaA, St. Louis, MO, USA) that has been supplemented with a protease inhibitor cocktail (104 mM AEBSF, 80 µM aprotinin, 4 mM Bestatin, 1.4 mM E-64, 2 mM leupeptin, and 1.5 mM pepstatin A; Sigma-Aldrich Corp., St. Louis, MO, USA) was used. The lysates were cleared by centrifugation at 10,000× *g* for 20 min, at 4 °C. Total protein concentrations were quantified using the bicinchoninic acid assay (BCA) method. Subsequently, each sample was loaded onto a Mini-PROTEAN^®^ TGX Stain-Free™ polyacrylamide gel (Bio-Rad, Hercules, CA, USA), and electrophoresed at a constant of 140 V for 30–40 min. The gels were then transferred onto a Trans-Blot^®^ Turbo™ Mini polyvinylidene difluoride (PVDF) membrane using the Trans-Blot^®^ Turbo™ Blotting System (Bio-Rad Laboratories, Hercules, CA, USA), with a pre-set protocol known as Mixed MW (1.3 A, 25 V, 7 min). The membranes were blocked using 5% *w/v* bovine serum albumin, heat shock fraction (Sigma-Aldrich Corp, St. Louis, MO, USA), for 1 h at room temperature in 1× Tris-buffered saline (TBST; 20 mM Tris, 150 mM NaCl, 0.1% Tween 20). Afterwards, the blots were probed with the primary antibodies overnight at 4 °C, except for GAPDH, which required only an hour of incubation. The following antibodies were used: NRAS (rabbit α-NRAS polyclonal antibody, Cat. No. PA5-34560; Invitrogen) and GAPDH (mouse α-GAPDH, Cat. No. CB1001; Calbiochem, San Diego, CA, USA). After incubation, the membranes were subjected to three washes with 1× TBST. Subsequently, they were incubated with a freshly prepared secondary antibody solution (diluted at 1:10,000 for both the antibody and Precision Protein™ StrepTactin-HRP conjugate from Bio-Rad) in the blocking solution for 1 h at room temperature. Band signals were visualized using an enhanced chemiluminescence substrate Luminata™ Classico Western HRP Substrate (Cat No. WBLUC0500; Merck Millipore, Burlington, MA, USA), and captured using the ChemiDoc Touch Imaging System (Bio-Rad Laboratories, Inc., Hercules, CA, USA).

### 2.4. Cell Proliferation Assay

At 24 h post-transfection, HCT116 and NIH3T3 cells were reseeded at a density of 3000 and 2500 cells per well, respectively, into separate 96-well plates, in triplicate. HCT116 cells were maintained in RPMI medium at a reduced serum concentration (4% FBS), while NIH3T3 cells were maintained in DMEM at a reduced serum concentration (4% NBCS). At 0, 48, and 72 h post-transfection, the number of metabolically active cells in each setup was determined by adding 10 µL of CellTiter 96^®^ Aqueous One-Cell Proliferation Assay Reagent (Cat. No. G358C; Promega, Madison, WI, USA) to each well. The plates were then incubated at 37 °C with 5% CO_2_ for 2 h. Absorbance values for each setup were subsequently measured at 460 nm using the ClarioStar multimode plate reader (BMG Labtech, Offenburg, Germany).

### 2.5. 3D Spheroid Assay

At 24 h post-transfection, HCT116 cells were reseeded from a 12-well plate into a black 96-well, round-bottom, ultra-low attachment spheroid plate (Cat. No.4520; Corning, Merck KGaA, Darmstadt, Germany), at a density of 3000 cells per well in triplicate. Once the spheroids had formed after 72 h, 50% of the media was removed, and 50 µL of CellTiter-Glo 3D Cell Viability Reagent (Cat No. G9681, Promega, Madison, WI, USA) was added. The plate was shaken vigorously using a plate reader shaker for 5 min, then incubated for 25 min at room temperature. Luminescence was read at 490 nm. In a parallel setup, high-content imaging using an IN Cell Analyzer 6000 (GE Healthcare, Marlborough, MA, USA) was employed to capture spheroid images. Once the spheroids had formed after 72 h, 50% of the media was removed, and 50 µL of fresh RPMI media containing 0.5 µM calcein AM (Cat. No. C3100MP; Thermo Fisher Scientific, Waltham, MA, USA) and 10 µM of DAPI per well was added. The plate was then incubated for 1 h at 37 °C in a 5% CO_2_ incubator.

### 2.6. Caspase 3/7 Assay

At 24 h post-transfection, transfected HCT116 cells were reseeded at a density of 10,000 cells per well, while transfected NIH3T3 cells were reseeded at 8000 cells per well, then allowed to attach overnight. This procedure was carried out in triplicate using separate 96-well plates (Cat. No. 3599; Corning, Corning, NY, USA) for each cell line. When the cells had attached after 24 h, the respective media were changed. HCT116 cells were cultured in RPMI medium with a reduced serum concentration (2.5% FBS), supplemented with 10 mM sodium butyrate, while NIH3T3 cells were maintained in DMEM medium with a reduced serum concentration (0.1% NBCS). Additionally, a parallel setup was performed, designated as the ‘uninduced setup’ because it lacked the apoptotic inducer sodium butyrate in the media. The plate was covered with aluminum foil and incubated at 37 °C with 5% CO_2_ for 16 h. After incubation, 20 µL of the Caspase-Glo^®^ 3/7 Reagent (Promega) was added to each well, and the plate was gently agitated for 2.5 h in the dark. Subsequently, the supernatant was transferred to a 96-well flat-bottom white plate (Cat. No. 3917; Corning), and read using a luminescence reader (Clariostar; BMG Labtech, Offenburg, Germany) at 530 nm. To measure apoptosis, the luminescence readings obtained from the induced setup were normalized against those from the uninduced setup, allowing for comparisons across the different setups.

### 2.7. Wound Healing Assay

At 24 h post-transfection, HCT116 cells were reseeded at a density of 35,000 cells per well, while transfected NIH3T3 cells were reseeded at 18,000 cells per well. Cells were then allowed to attach overnight. Once the cells were 80–90% confluent, a thin, artificial scratch was created in the cell monolayer using a sterile white micropipette tip. Subsequently, the medium was replaced with a low-serum medium, which consisted of RPMI with 2.5% FBS for HCT116 cells, or DMEM with 2.5% NBCS for NIH3T3 cells. Both cell lines were incubated at 37 °C with 5% CO_2_, and wound closure was monitored by capturing images of the same field of view both immediately after scratching and 16 h post-scratch, using the IN Cell Analyzer 6000 (GE Healthcare) high-content imaging system. The percentage of the open wound area was analyzed using the TScratch software for automated analysis of monolayer wound healing assays [30]. For each setup, a linear graph illustrating the percentage of the open wound area over time was generated, and the slope of each line equation was calculated to determine the rate of wound closure. All experiments were conducted three times, each in triplicate.

### 2.8. Actin Cytoskeletal Staining

The impact of the novel NRAS mutants—G48C, Q43K, and E37K—on cytoskeletal organization was assessed via actin staining with Alexa Fluor™ 488-conjugated phalloidin (Invitrogen). Transfected NIH3T3 cells were seeded at 2500 cells/well in a 96-well black plate. After 24 h, cells were fixed with ice-cold 4% paraformaldehyde for 20 min, with shaking at 40 rpm on ice. Following a rinse with 1× PBS, the cells underwent a 20 min blocking step at room temperature, using 1% BSA in PBS. Subsequently, they were incubated with 100 µL of 0.165 µM phalloidin for 1 h at room temperature while gently shaking. The cells were subjected to another round of washing with 1× PBS before counterstaining the nuclei with Hoechst 33,258 (1 µg/µL) for a 5 min incubation at room temperature. Following the last wash with 1× PBS, the cells were mounted using SlowFade^TM^ Diamond antifade mountant (Invitrogen). Cells were visualized using the IN Cell Analyzer 6000 (GE Healthcare) high-content imaging system, from multiple randomized perspectives.

### 2.9. Protein Modeling and in Silico Prediction of the Functional Impact of the Novel NRAS Mutations

To evaluate the effects of the identified single amino acid mutations on protein structure, models for NRAS G48C, Q43K, and E37K were generated using SWISS-MODEL [31] (accessed through https://swissmodel.expasy.org (accessed on 12 July 2024)). Root mean square deviation was calculated by superimposing the generated models with the wild-type structure (PDB ID: 5UHV), using PyMOL v4.6 (https://www.pymol.org; (accessed on 12 July 2024)). Predictions of the potential functional impact of the NRAS missense mutations were assessed using the following: Polymorphism Phenotyping (PolyPhen-2 version 2) [32], PROVEAN software (Protein Variation Effect Analyzer v1.1) [33], Align GVGD [34,35], MutPred 2.0 [36], and FATHMM v2.3 [37]. For GDP docking simulations, the AutoDock Vina 1.5.7 package was used [38]. Briefly, pdbqt files were prepared for all homology models and GDP, which were used as the receptors and ligand, respectively. AutoDock Vina was then run in the command prompt, and the resulting docked configurations were visualized using PyMOL (The PyMOL Molecular Graphics System, Version 3.0, Schrödinger, LLC, Boston, MA, USA).

### 2.10. Statistical Analysis

All experiments were conducted at least three times, and each trial involved at least three technical replicates. To compare means from three or more groups, analysis of variance (ANOVA) followed by Tukey’s HSD post hoc test was used to compare differences among multiple setups. Results from all quantitative experiments were presented as mean ± standard deviation. In all statistical tests, significance was defined as follows: * for *p* < 0.05; ** for *p* < 0.01; *** for *p* < 0.001; and **** for *p* < 0.0001.

## 3. Results

### 3.1. NRAS G48C, Q43K, and E37K Promote Cellular Proliferation in HCT116 and NIH3T3 Cells

**Figure 1:**
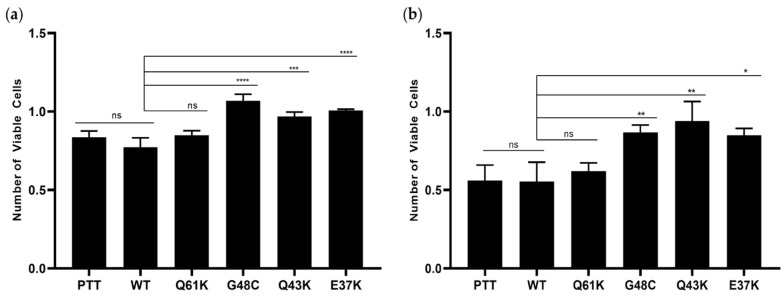
Effect of the novel NRAS mutants on cell proliferation. NRAS G48C, Q43K, and E37K enhanced proliferation of both HCT116 and NIH3T3 cells compared to vector-only, wild-type, and canonical mutant control Q61K. Proliferation rates of (**a**) HCT116 cells and (**b**) NIH3T3 cells transfected with empty vector, wild-type NRAS, canonical NRAS Q61K mutant, and the novel NRAS mutants G48C, Q43K, and E37K are shown. Data presented are representative of three independent trials, each performed in triplicate, and expressed as mean ± standard deviation (SD). * *p* ≤ 0.05, ** *p* ≤ 0.01, *** *p* ≤ 0.001, **** *p* ≤ 0.0001. PTT = pTargeT vector-only control; WT = wild-type; ns = non-significant.

To assess the impact of the novel NRAS G48C, Q43K, and E37K mutations on the proliferative capacity of HCT116 and NIH3T3 cells, the CellTiter 96^®^ AQueous One Solution Cell Proliferation Assay was carried out. Cell counts were conducted over three consecutive days. All novel mutants enhanced cell proliferation, with a notable increase in cell count at 72 h post-transfection (Figure 1a,b) compared to the vector-only, wild-type, and canonical mutant control NRAS Q61K, and they deviated from previous observations that mutations in NRAS do not affect cellular proliferation [16,21,39].

### 3.2. NRAS G48C, Q43K, and E37K Promote 3D Spheroid Formation in HCT116 Cells

**Figure 2:**
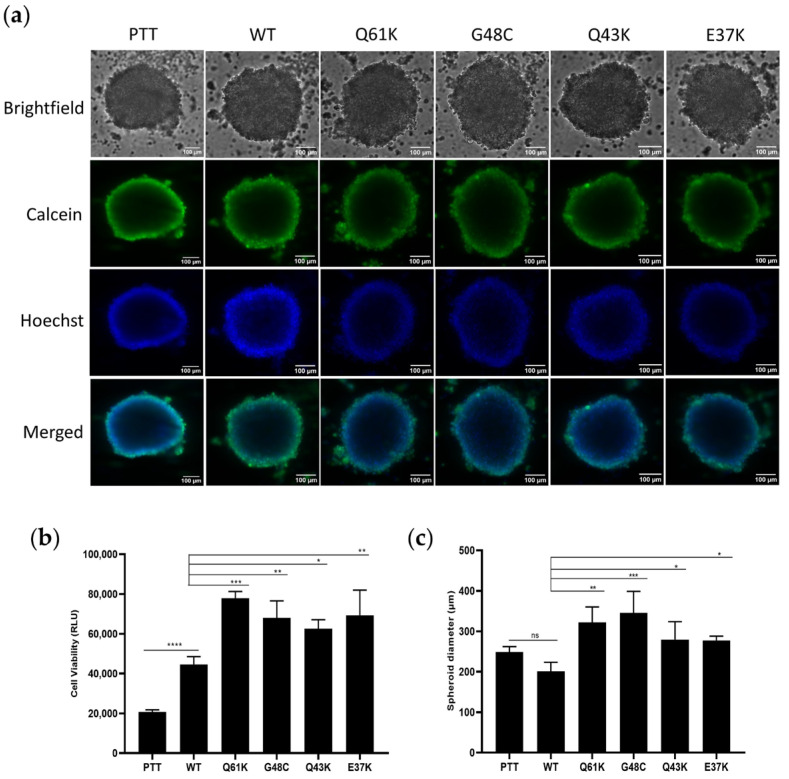
3D spheroid cultures transfected with pTT, WT, NRAS Q61K, and novel NRAS mutants. (**a**) Images of spheroids for all of the constructs. Spheroids were stained with calcein and Hoechst to visualize spheroid formation. (**b**) Quantification of cell viability (RLU values). (**c**) Measurement of 3D spheroid diameter. Data presented are representative of three independent trials, each performed in triplicate, and expressed as mean ± standard deviation (SD). * *p* ≤ 0.05, ** *p* ≤ 0.01, *** *p* ≤ 0.001, **** *p* ≤ 0.0001. PTT = pTargeT vector-only control; WT = wild-type; ns = non-significant.

To assess the reproducibility of the observed results in a model that approximates the spatial organization of tumor cells, HCT116 cells transfected with constructs of the NRAS mutants, along with the wild-type, vector-only, and NRAS Q61K canonical mutant controls, were cultured as 3D spheroids. Mere overexpression of wild-type NRAS promoted the proliferation of cells compared to the vector-only control, but the canonical mutant Q61K and the novel mutants G48C, Q43K, and E37K further promoted cellular proliferation compared to the wild-type (Figure 2b,c) in CellTiter-Glo™ 3D Cell Viability Assays. The representative images (Figure 2a) confirm the ability of the mutants to promote cellular proliferation in a more physiologically relevant model compared to 2D assays.

The relative luminescence units (RLU) and tumor spheroid diameters were measured (Figure 2a,b). The RLU values in the context of the 3D spheroid assay are measures of the luminescent signal produced by spheroid structures, and they are used to quantify cell viability. If RLUs are higher in cancer cells, this could indicate increased metabolic activity, enhanced proliferation, increased viability, or reduced apoptosis. The ATP levels of metabolically active 3D cultures correlate with the range of luminescence outputs observed in the assay [40]. Significant compression of tumor spheroids amplifies cancer cell proliferation and metastatic process [41].

### 3.3. The Novel NRAS Mutants G48C, Q43K, and E37K Promote Apoptosis Resistance

**Figure 3:**
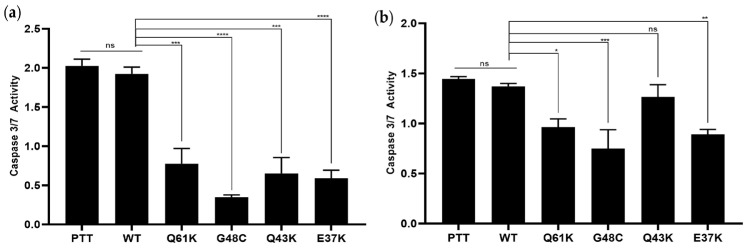
Effect of the novel NRAS mutants, G48C, Q43K, and E37K, on cell survival. (**a**) HCT116 and (**b**) NIH3T3 cells were transfected with NRAS WT and mutant constructs, then assessed for their ability to resist apoptosis when induced with sodium butyrate (HCT116) and reduced serum concentration (NIH3T3). Data presented are representative of three independent trials, each performed in triplicate, and expressed as mean ± standard deviation (SD). * *p* ≤ 0.05, ** *p* ≤ 0.01, *** *p* ≤ 0.001, **** *p* ≤ 0.0001. PTT = pTargeT vector-only control; WT = wild-type; ns = non-significant.

The novel NRAS mutants G48C, Q43K, and E37K, alongside the vector-only, wild-type NRAS, and canonical Q61K mutant controls, were evaluated for their ability to inhibit apoptosis in HCT116 and NIH3T3 cells (Figure 3a,b, respectively) via the Caspase-Glo 3/7 assay. The novel NRAS mutants, G48C and E37K, showed significant resistance to apoptosis, similar to the canonical mutant Q61K, and in contrast to the wild-type and vector-only controls in both HCT116 and NIH3T3 cells. However, Q43K showed resistance only in HCT116, and not in NIH3T3 cells. Overall, these findings are consistent with the previous literature on NRAS, showing that mutations in the oncogene can lead to apoptosis resistance [16,21,42].

### 3.4. NRAS G48C, Q43K, and E37K Show No Effect on Cellular Migration in NIH3T3, but G48C Enhanced the Migration Rate of HCT116 Cells

**Figure 4:**
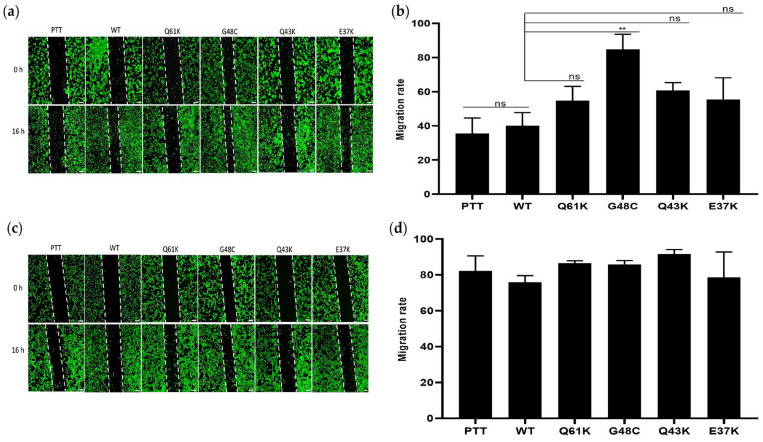
Effect of NRAS G48C, Q43K, and E37K mutants on cellular migration in HCT116 and NIH3T3 cells. Scratch wound assay showing representative images of (**a**) HCT116 and (**c**) NIH3T3 cells transfected with empty vector, wild-type NRAS, canonical NRAS Q61K control, and the novel NRAS mutants G48C, Q43K, and E37K at 0 h and 16 h post-scratch. The migration rates for each setup are shown in (**b**,**d**) for HCT116 and NIH3T3 cells, respectively. Data presented are representative of three independent trials, each performed in triplicate, and expressed as mean ± standard deviation (SD). ** *p* ≤ 0.01. pTT = pTargeT vector-only control; WT = wild-type; ns = non-significant.

Scratch wound assays were conducted to assess the impact of the novel NRAS mutants on the migratory capacity of both HCT116 and NIH3T3 cells. NRAS G48C enhanced the migration rate of HCT116 cells compared to wild-type and vector-only controls, particularly in conditions of reduced serum concentration. Similar to the canonical NRAS mutant Q61K, Q43K and E37K did not show a significant difference in the migration rate when compared to the wild-type and vector-only controls (Figure 4a,b).

Cells transfected with the canonical and three novel NRAS mutants exhibited no effect on the wound closure rate in NIH3T3 cells (Figure 4b,d). Earlier investigations on the canonical NRAS G12D and Q61K mutants, as well as the novel NRAS mutant E132K, did not show any effect on cell migration in either HCT116 and NIH3T3 cells [21].

### 3.5. NRAS G48C, Q43K, and E37K Induce Cytoskeletal Remodeling in NIH3T3 Cells

**Figure 5:**
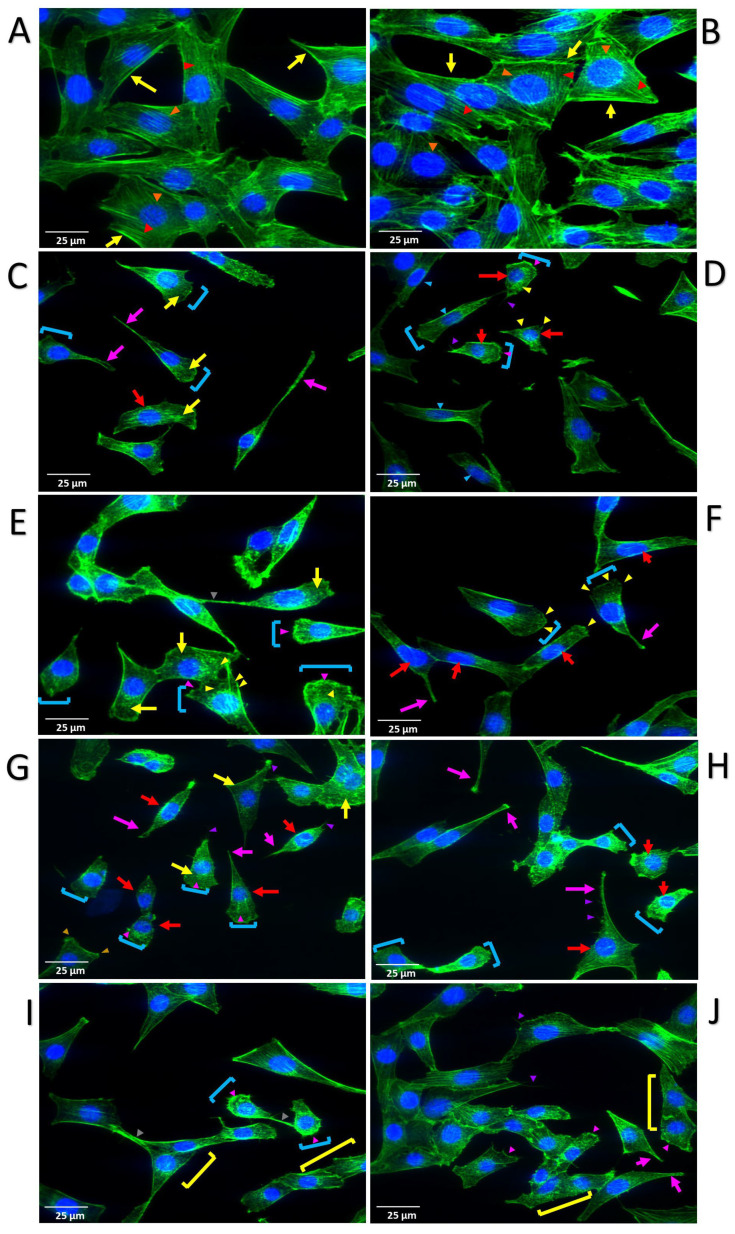
Expression of NRAS mutants induce cytoskeletal remodeling in NIH3T3 cells. Distinct features, such as prominent peripheral stress fibers (yellow arrows) and highly organized actin filaments (red arrowheads), are present in both the empty vector (**A**) and wild-type (**B**) setups. Central round nuclei (orange arrowheads) are also visible in WT. Changes in cytoskeletal architecture, as well as invasive structures, are observable in both canonical and novel mutant setups (**C**,**D** = Q61K; **E**,**F** = G48C; **G**,**H** = Q43K; **I**,**J** = E37K), including fan-shaped cells with prominent migrating front (sky blue brackets); peripheral dorsal ruffles (pink arrowheads); circular dorsal ruffles (yellow arrowheads); tunneling nanotubes (gray arrowheads); multilobulated nuclei (red arrows); invadopodia (pink arrows); lamellipodia (gold arrowheads); filopodia (purple arrowheads); and fusing cells (yellow brackets).

The effects of the novel NRAS mutants on cytoskeletal organization were investigated by staining with Alexa Fluor™ 488-conjugated phalloidin, which visualizes F-actin and Hoechst 33,342 for nuclear staining (Figure 5). Across all mutant variants, distinctive alterations were observed, including the presence of hair-like filopodia and actin-rich lamellipodia; a reduction in cytoplasmic volume, resulting in shrunken and punctate cytoplasm; the formation of thick peripheral actin structures, including peripheral and circular dorsal ruffles; the presence of multilobulated nuclei and fusing cells that are associated with tumorigenesis; and the emergence of tunneling nanotube-like formations. Concurrently, protrusive invadopodia were also identifiable in some cells, collectively suggesting a comprehensive restructuring of the cytoskeleton that is indicative of heightened motility and invasive potential. In contrast, cells transfected with wild-type NRAS exhibited well-organized parallel stress fibers, with no significant morphological changes observed, except for a denser appearance compared to pTargeT vector-only controls. Similar to other, previously described cancer phenotypes, this finding aligns with prior studies, indicating that the mere overexpression of wild-type RAS variants is inadequate to induce a transformed cellular phenotype in NIH3T3 cells [21,43].

### 3.6. Prediction of the Oncogenic Impact of NRAS Mutants through Bioinformatics-Based Modeling and Molecular Docking Simulations

**Figure 6:**
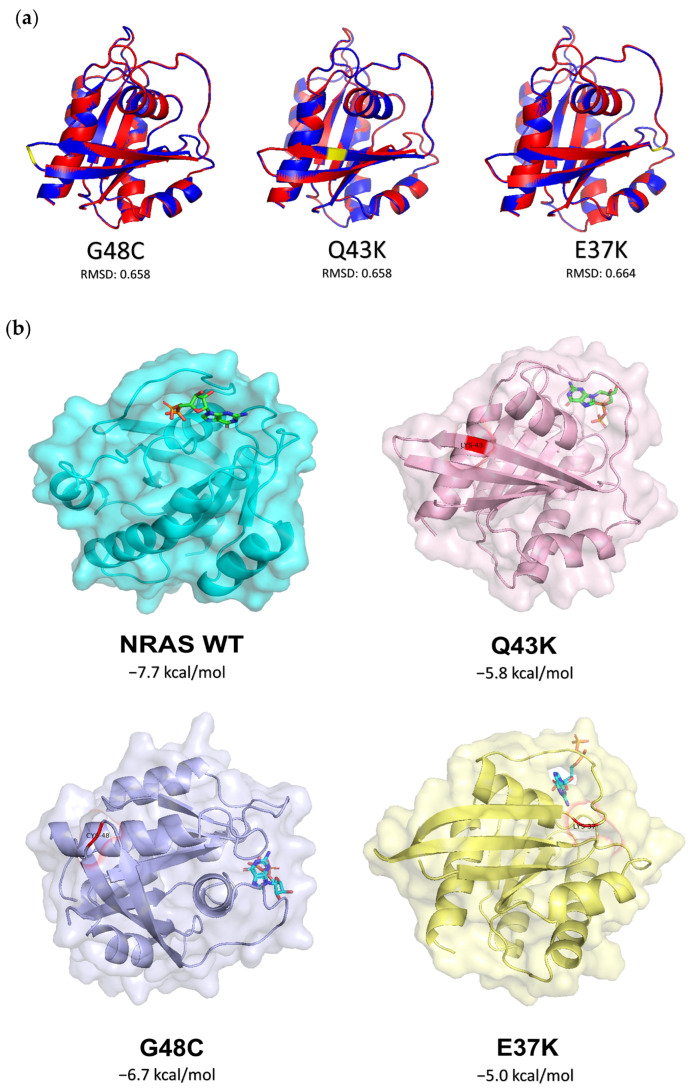
In silico analyses of the potential oncogenic impact of the novel NRAS mutants G48C, Q43K, and E37K. (**a**) Homology modeling of NRAS mutants suggests potential oncogenic impact. NRAS is depicted in blue, with its mutated residue highlighted in yellow, superimposed on wild-type NRAS, which is shown in red. (**b**) AutoDock Vina Protein-Ligand Docking. Generated homology structures of the NRAS mutants were used as receptors to dock GDP ligands. The mutated residues of each mutant are shown in red. The docked configurations shown represent GDP-binding to the nucleotide-binding pocket, as seen in the initially published structure (NRAS WT; PDB ID: 5UHV).

Protein in silico analysis was conducted for NRAS G48C, Q43K, and E37K to assess the structural changes induced by the missense mutations. Generated homology models (Figure 6a) were compared to those of NRAS wild-type (PDB ID: 5UHV), and the resulting RMSDs are 0.658 Å, 0.658 Å, and 0.664 Å for G48C, Q43K, and E37K, respectively. The RMSD value represents the average distance between the alpha-carbon backbones of the analyzed models, with a higher value indicating a greater predicted deviation between the molecules [44,45]. This suggests that the NRAS missense mutations may not have substantially altered the structure of the wild-type protein, which is expected of single nucleotide variations. To further assess the effect of the NRAS mutants, web-based prediction tools were used. These rely on the evolutionary conservation of the amino acid sequence, the biochemical properties of each residue, and a recorded deleterious effect on known diseases [32,33,34,35,36,37]. NRAS G48C, Q43K, and E37K were all predicted to be deleterious or pathogenic (Table 2), despite inducing minimal changes in structure. So far, these results suggest that the functional consequences of single amino acid substitutions do not solely rely on the overall protein structure.

To probe the functional aspect of NRAS, GDP docking simulations were performed using AutoDock Vina [38]. Nucleotide hydrolysis and exchange dictate the activation state of Ras proteins; therefore, alterations in this mechanism can either promote or inhibit oncogenicity. In this study, nucleotide exchange was assessed by identifying the binding affinity of GDP to the binding site of NRAS. As shown in the resulting docked configurations (Figure 6b), only in the wild-type structure was GDP able to bind fully within the binding pocket. The phosphate group of GDP is notably protruding outward of the binding pocket for all of the NRAS mutants, unlike in the wild-type version. Moreover, comparing the GDP-binding affinities, NRAS G48C, Q43K, and E37K all have less negative ΔG values, indicating a weaker binding affinity compared to the wild-type version. These results suggest that the missense mutations can affect GDP-binding, and potentially nucleotide exchange, thus corroborating the predicted functional and oncogenic impact.

## 4. Discussion

As sequencing initiatives maximize the advantages offered by next-generation sequencing—which allows for comprehensive gene coverage—the discovery of uncharted novel mutations outside mutational hotspots will become more common. While many of them are likely to be infrequent, the American Society for Clinical Oncology posits that rare mutations, including those in KRAS, may represent new mechanisms of resistance, and therefore argued for expanded KRAS and NRAS mutational analysis [46,47]. Tumor heterogeneity is another reason for cataloguing mutations that may coexist in cancer tissues. Pockets of cancer cells harboring rare mutations that confer resistance may survive initial therapeutic intervention and become the predominant cells in the event of a relapse. In the context of clinical management, ethnic nuances in the mutational profiles of cancer patients across the globe may further add to the already complex challenges posed by tumor heterogeneity.

Our prospective, targeted, next-generation sequencing study of *EGFR* pathway genes in tumors obtained from Filipino young-onset colorectal cancer patients [20] confirmed extensive inter- and intratumor heterogeneity, and identified dozens of novel coding region mutations, many of which have not been reported in the literature, or remain only partially characterized. In addition to the three NRAS mutants described in this study, other novel mutations in KRAS, NRAS, RRAS, BRAF, PIK3CA, PTEN, and MEK1 are still undergoing detailed characterization and analysis, and are currently unpublished. Here, the three novel NRAS mutations—c.142G>T (G48C), c.127C>A (Q43K), and c.109G>A (E37K)—were characterized for their effects on cancer phenotypes in vitro, using NIH3T3 and HCT116 cells. NIH3T3 is a preferred cell line for characterizing RAS oncogenes and their mutant variants, because of its wild-type background, and the fact that it does not require complementation of cooperative oncogenes for the effect of a transfected mutant construct to manifest [23,24,25,26,27,48,49], except, perhaps, for assays such as those for epithelial-to-mesenchymal transition that require an epithelial background. The most commonly used CRC cell lines, on the other hand, have variable background mutations in either KRAS, PIK3CA, or BRAF that may confound the interpretation of the phenotypes observed. To wit, SW480 has a KRAS G12V mutation; SW948 has KRAS Q61L and PIK3CA E542K; DLD-1 has G13D; HT29 has BRAF V600E; and HCT116 has KRAS G13D and PIK3CA H1047R [50]. This does not yet include the mutational status of some of these cell lines for adenomatous polyposis coli (APC) and p53. Hence, the impact of introduced mutations may be masked by, and may be difficult to decouple from, the effects of the variable background mutations in different cellular environments. Although harboring endogenous KRAS G13D and PIK3CA H1047R mutations, the oncogenic phenotypes of HCT116 may still be modulated in most cases via overexpression of oncogenes and their mutant variants; furthermore, the effects of the latter are decoupled from those of background mutations through the use of relevant controls [51], although this is not always the case. Unlike KRAS mutations, which are very well characterized in CRC for their clinical consequences, as well as for their molecular and cellular effects on individual cancer hallmarks, there are fewer studies on NRAS mutants, most of which only report on their identification and their impact on lack of response to anti-EGFR therapy. To date, the functionally characterized NRAS mutants are limited mostly to codons 12, 13, and 61 [19,21,52,53,54,55,56]. More recently, A11V and E132K have been added to this finite list of characterized NRAS mutants [21,36].

In this study, cells expressing the novel NRAS mutants, G48C, Q43K, and E37K, all exhibited an enhanced proliferative capacity in both HCT116 and NIH3T3 cells. This was corroborated by the results of spheroid assays, which replicate specific aspects of 3D growth and enhance cell–cell interaction to provide a more relevant simulation of tumor physiology [57]. NRAS G48C, Q43K, and E37K showed significantly higher RLU values in 3D cell viability assays compared to wild-type and vector-only controls, which is indicative of enhanced proliferation. Further, cells expressing G48C, Q43K, and E37K produced spheroids with a larger diameter than wild-type and vector-only controls. This is in contrast to previous studies on NRAS mutants, which showed no apparent effect on cell proliferation both in vitro and in animal models [16,21,39,42]. Notably, mice carrying a Cre-dependent activated allele of NRAS (G12D) did not induce hyperproliferation, while KRAS G12D did, which manifested as chronic intestinal hyperplasia in the colonic epithelium [39]. It is interesting to note, however, that cells expressing the NRAS Q61K canonical mutant showed enhanced proliferation in 3D spheroids, but had no apparent effect on proliferation in 2D cultures. This suggests that 3D physical architecture and enhanced cell–cell interactions may influence the phenotypic readout of individual mutations.

Similar to the Q61K canonical mutant control, the NRAS mutants G48C and E37K conferred resistance to apoptosis in both HCT116 and NIH3T3 cells, as indicated by a significant decrease in caspase 3/7 activity. The NRAS mutant Q43K, on the other hand, had no anti-apoptotic effect on NIH3T3 cells. The reason for this differential effect of Q43K on the two cell lines is not immediately apparent. It is plausible that Q43K may not really have an anti-apoptotic effect, and that the level of caspase 3/7 activity seen for the Q43K setup in HCT116 cells can be solely attributed to the endogenous PIK3CA H1047R mutation in the cell line. In contrast, G48C, E37K, and the canonical Q61K control showed decoupled anti-apoptotic effects in the wild-type background of NIH3T3 cells. The results for NRAS G48C and E37K, but not for Q43K, are congruent with both in vitro and in vivo studies, showing that while KRAS mutations do not have pro-survival effects, NRAS mutants do [16,21,39,42].

Neither the three novel NRAS mutants nor the canonical NRAS Q61K mutant promoted cell migration in NIH3T3 cells. NRAS G48C, however, significantly enhanced cellular migration in HCT116 cells, even in reduced serum conditions. It is possible that the pro-migratory effect of NRAS G48C only manifested in the presence of the endogenous KRAS G13D mutation in HCT116. Such a context-dependent requirement for a cooperative mutation is not without precedent in atypical oncogenic variants. For instance, the kinase-dead BRAF D594F mutation is able to hyperactivate ERK phosphorylation in the presence of concurrent RAS mutations, yet it lacks such an activity when RAS is wild-type [58]. Similar to previously characterized NRAS mutants, including G12D, G12V, Q61K, A11V, and E132K, the novel NRAS Q43K and E37K mutants showed no effect on cellular migration [21,42,59].

F-actin cytoskeletal staining of NIH3T cells expressing the NRAS mutants revealed morphological alterations indicative of cytoskeletal remodeling. All novel mutant setups, as well as cells expressing the canonical Q61K mutant, displayed a shrunken cytoplasm with a punctate actin staining characteristic of cytoskeletal disassembly. In contrast, cells transfected with the vector-only control or the wild-type NRAS construct were generally larger, polygonal in shape, and have highly organized parallel stress fibers. As well, cells expressing the mutant constructs were mostly detached or solitary, and many are fan-shaped, with a prominent leading edge. Transient structures associated with motility and invasiveness were also apparent in all mutant setups. Many nuclei are surrounded by contractile actin bundles known as perinuclear actin rims, which help flatten or stretch nuclei to facilitate cellular migration while preserving genome integrity [60,61]. Invadopodia, which help degrade the extracellular matrix [62], are also identifiable. Some cells display peripheral dorsal ruffles, as well as circular dorsal ruffles which have collapsed inwards to soften the cytoskeleton [63]. Tunneling nanotubes (TNTs) extending from cell to cell are identifiable in cells transfected with G48C and E37K. TNTs have been shown to facilitate intercellular transport of RNA transcripts, proteins, and even organelles such as mitochondria, to instigate an oncogenic program or promote survival of damaged cells [64,65]. Lastly, cell fusion—a result of aberrant mitosis sometimes observed in some malignancies [66]—can be observed in cells transfected with NRAS E37K.

Finally, the in silico analysis carried out predicted a deleterious or pathogenic consequence for each of the NRAS mutants. While this study carried out transient overexpression experiments, the oncogenic effects of NRAS G48C, Q43K, and E37K are evident in different cancer hallmark assays. It is also noteworthy that RMSD calculations have an intrinsic limitation of only analyzing rigid structures; nonetheless, single amino acid substitutions are not expected to substantially affect the overall protein structure. However, ligand docking simulations, along with the resulting functional predictions, imply that the changes in overall protein structure do not necessarily translate to an immediate change in function. Furthermore, in relation to the results of in vitro experiments, it is intriguing to speculate whether an altered or increased nucleotide exchange could explain the functional consequences observed. While much of the literature on this mechanism is focused on the other RAS isoforms, the results of initial docking simulations warrant further investigation to better understand the oncogenic program prompted by the NRAS mutants in CRC.

## Figures and Tables

**Table 1:** *NRAS* Primer Sequences.

Title 1	Sequence (5’-3’) *
G48C-ext-fwd	TATCGCTCGAGATGACTGAGTACAAACTGGTGGTGGTTGGAG
G48C-mut-rev	TGTCCAACAAACAGGTTTCAC**A**ATCTATAACCACTTG
G48C-mut-fwd	ACAAGTGGTTATAGAT**T**GTGAAACCTGTTTGTTGGAC
G48C-ext-rev	TCTAGGTCGACACACATGGCAATCCCATACAACCTTACATCACC
Q43K-InvPCR-fwd	GGATTCTTACAGAAA**A**AAAGTGGTTATAGATGGTGAAACCTG
Q43K-InvPCR-rev	CCATCTATAACCACTT**T**TTTTCTGTAAGAATCCTCTATGGTG
E37K-InvPCR-fwd	GATCCCACCATA**A**AGGATTCTTACAGAAAACAAGTGGT
E37K-InvPCR-rev	GTTTTCTGTAAGAATCCT**T**TATGGTGGGATCATATTCATCTACA

(*) Nucleotide in bold font denotes the mutated nucleotide.

**Table 2:** Assessment of NRAS missense variants using web-based prediction tools.

	PROVEAN	Polyphen-2	ALIGN GVGD	MutPred	FATHMM
G48C	Deleterious (−3.606)	Probably damaging (0.998)	Highly pathogenic (Class65)	Pathogenic (0.850)	Cancer (−2.83)
Q43K	Deleterious (−6.339)	Possibly damaging (0.493)	Pathogenic (Class 45)	Pathogenic (0.864)	Cancer (−2.13)
E37K	Deleterious (−3.046)	Possibly damaging (0.506)	Highly pathogenic (Class 55	Pathogenic (0.788)	Cancer (−2.47)

## Data Availability

All data generated or analyzed in this study are available from the corresponding author upon reasonable request. Next-generation sequencing data, from which the novel NRAS mutants reported in this study were identified, are available via BioProject accession number PRJNA1156316.

## Supplementary Materials

The following supporting information can be downloaded at: https://www.mdpi.com/article/10.3390/cells13201691/s1, Figure S1: Confirmation of NRAS expression from the different gene constructs via Western blot analysis in (A) HCT116 and (B) NIH3T3 cells.
