# Supplementary material for: Atypical Exon 2/3 Mutants G48C, Q43K, and E37K Present Oncogenic Phenotypes Distinct from Characterized NRAS Variants
Source: Cells. 2024 Oct 12;13(20):1691. doi: 10.3390/cells13201691 (PMC11506670; doi:10.3390/cells13201691)
Supplement: Supplementary file 1 [file cells-13-01691-s001.zip › cells-3227833-supplementary.pdf]

Atypical exon 2/3 mutants G48C, Q43K, and E37K present oncogenic phenotypes distinct from characterized NRAS variants

Mark Anthony G. Fran [1], Dominique Mickai G. Leaño [2], James Allen D. de Borja [2], Charles John T. Uy [2,†], Aleq Adrianne R. Andresan [2], Dennis L. Sacdalan [2,3] and Reynaldo L. Garcia [2,*]

1 The Graduate School, Thomas Aquinas Research Complex, University of Santo Tomas, España, Manila 1008, Philippines; fran.biology@gmail.com
2 Disease Molecular Biology and Epigenetics Laboratory, National Institute of Molecular Biology and Biotechnology, University of the Philippines Diliman, Quezon City, 1101, Philippines; dgleano@up.edu.ph (D.M.G.L.); jamesallendeborja@gmail.com (J.A.D.D.); arandresan@up.edu.ph (A.A.R.A.)
3 Division of Medical Oncology, Department of Medicine, University of the Philippines, Manila City, 1000, Philippines; dlsacdalan1@up.edu.ph
* Correspondence: rlgarcia1@up.edu.ph
† Present Address: Olivia Newton-John Cancer Research Institute, and School of Cancer Medicine, La Trobe University, Heidelberg, VIC, 3084, Australia; charles.uy@onjcri.org.au

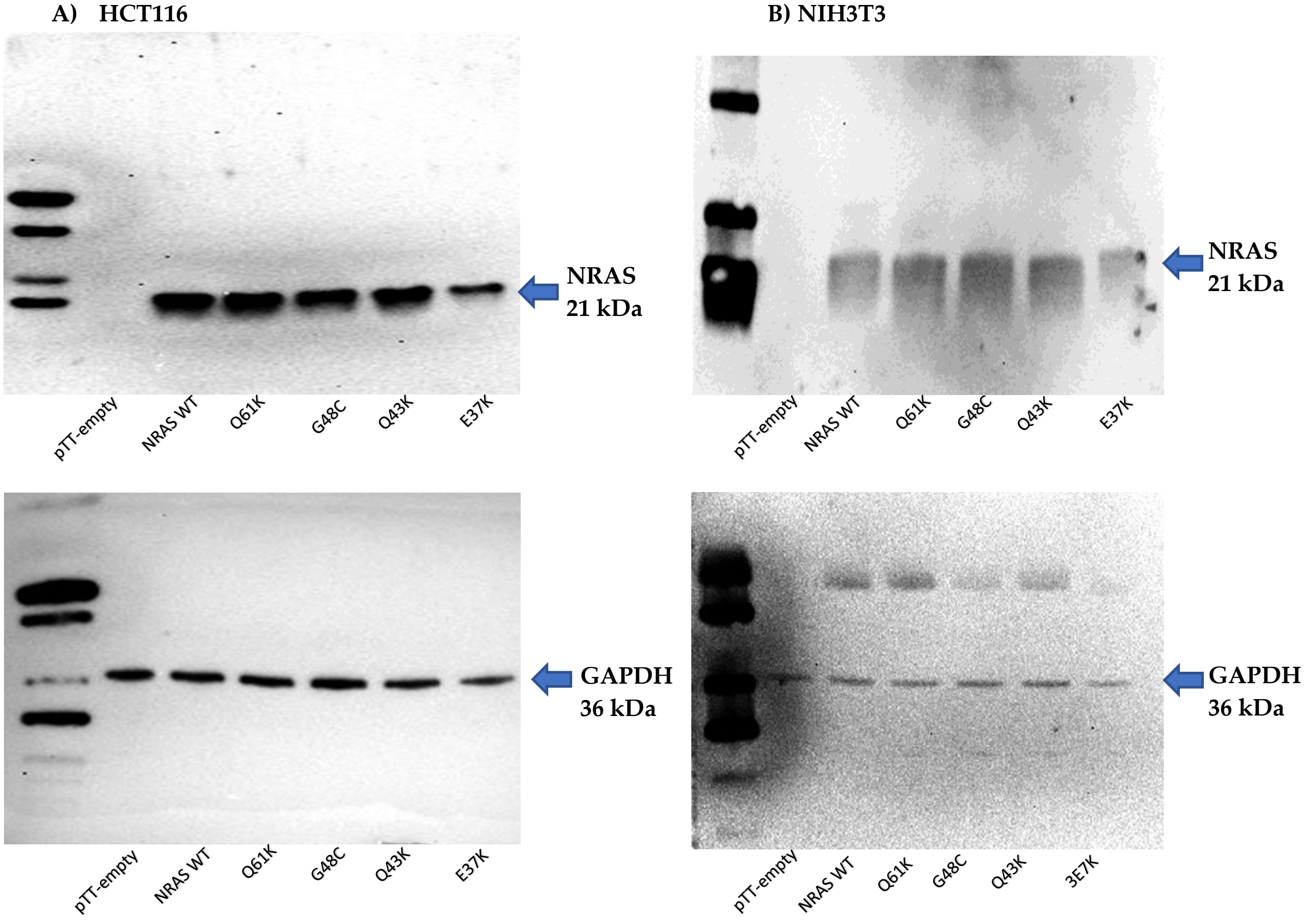

Figure S1. Confirmation of NRAS expression from the different gene constructs via Western blot analysis in A) HCT116 cells and B) NIH3T3. The blot was also probed for GAPDH which served as an internal loading control for normalization.
